# Acute-onset IgA pemphigus with rapid durable remission: A case supporting an infection-triggered reactive form

**DOI:** 10.1016/j.jdcr.2026.09.044

**Published:** 2026-09-30

**Authors:** Jules Roullée, Florian Lombart, Amine Moslemi, Guillaume Chaby, Théo Brochet

**Affiliations:** a Department of Dermatology, CHU Amiens-Picardie, Amiens, France; b Department of Pathology, CHU Amiens-Picardie, Amiens, France; c Department of Dermatology, GHPSO (Groupe Hospitalier du Sud de l’Oise), Creil, France

**Keywords:** IgA pemphigus, intraepidermal neutrophilic type, *Mycoplasma pneumoniae*, topical corticosteroids

## Introduction

IgA pemphigus is a rare autoimmune bullous dermatosis characterized by neutrophilic infiltration, acantholysis, and IgA deposit on the surface of keratinocytes. Two main variants are recognized: the subcorneal pustular dermatosis type and the intraepidermal neutrophilic (IEN) type. While dapsone is considered the first-line treatment, management is not standardized due to the rarity of the disease. We report a case of a possible *Mycoplasma pneumoniae*-induced IEN-type IgA pemphigus presenting with systemic symptoms and achieving rapid, sustained remission with high-potency topical corticosteroids alone.

## Case report

A 42-year-old woman of North African descent with no significant past medical history presented with a sudden, highly inflammatory eruption. Clinical examination revealed vesicles and bullae, overlying a violaceous erythematous background on the lower limbs (Fig 1). Small, disseminated pustules and larger vesicles were noted on the upper limbs (Figs 2 and 3). No mucosal involvement was noted, and the patient reported no recent drug intake.

**Fig 1:**
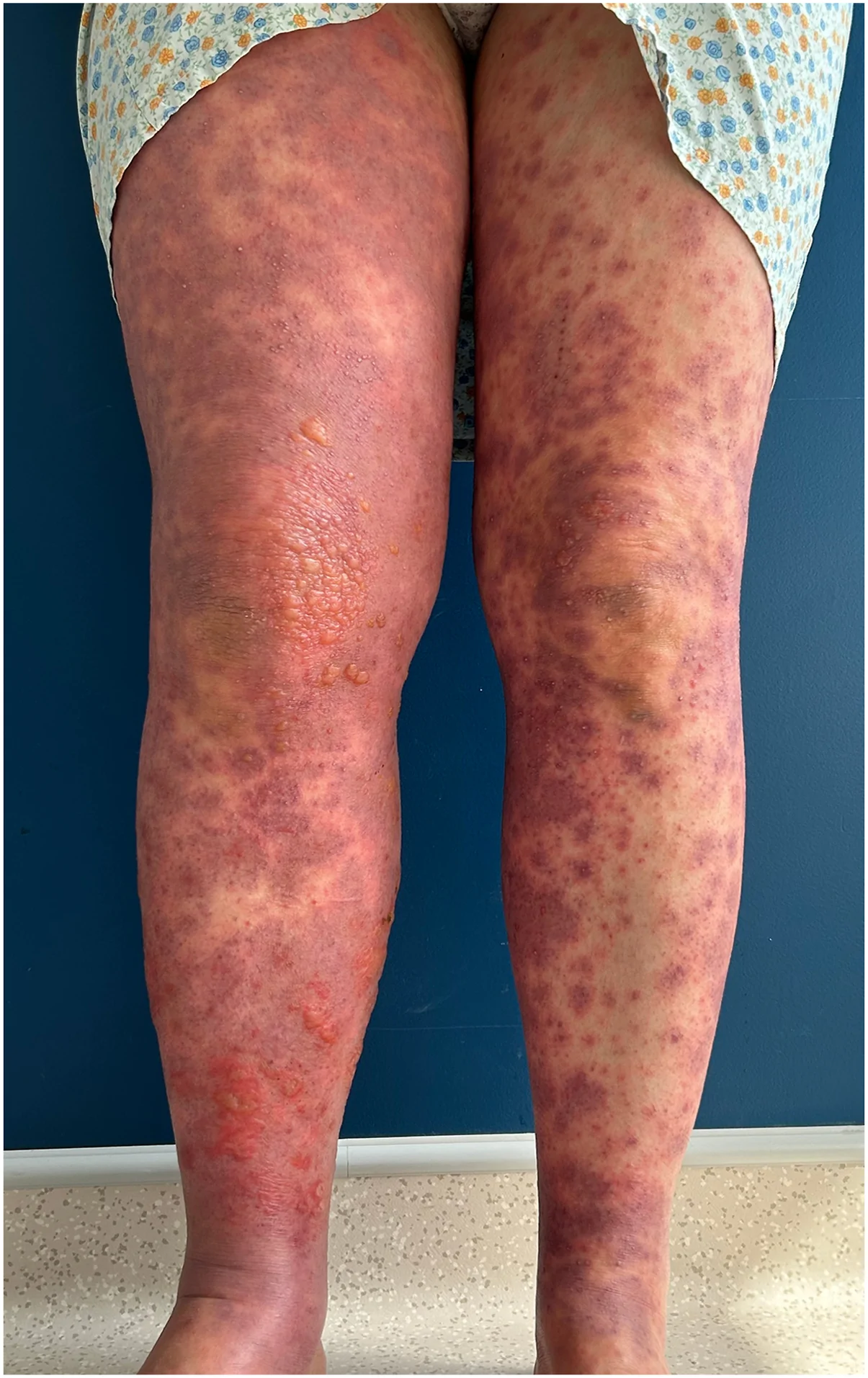
Clinical presentation of the lower limbs showing a highly inflammatory and infiltrated eruption. Note the presence of multiple vesicles and bullae overlying a violaceous erythematous background.

**Fig 2:**
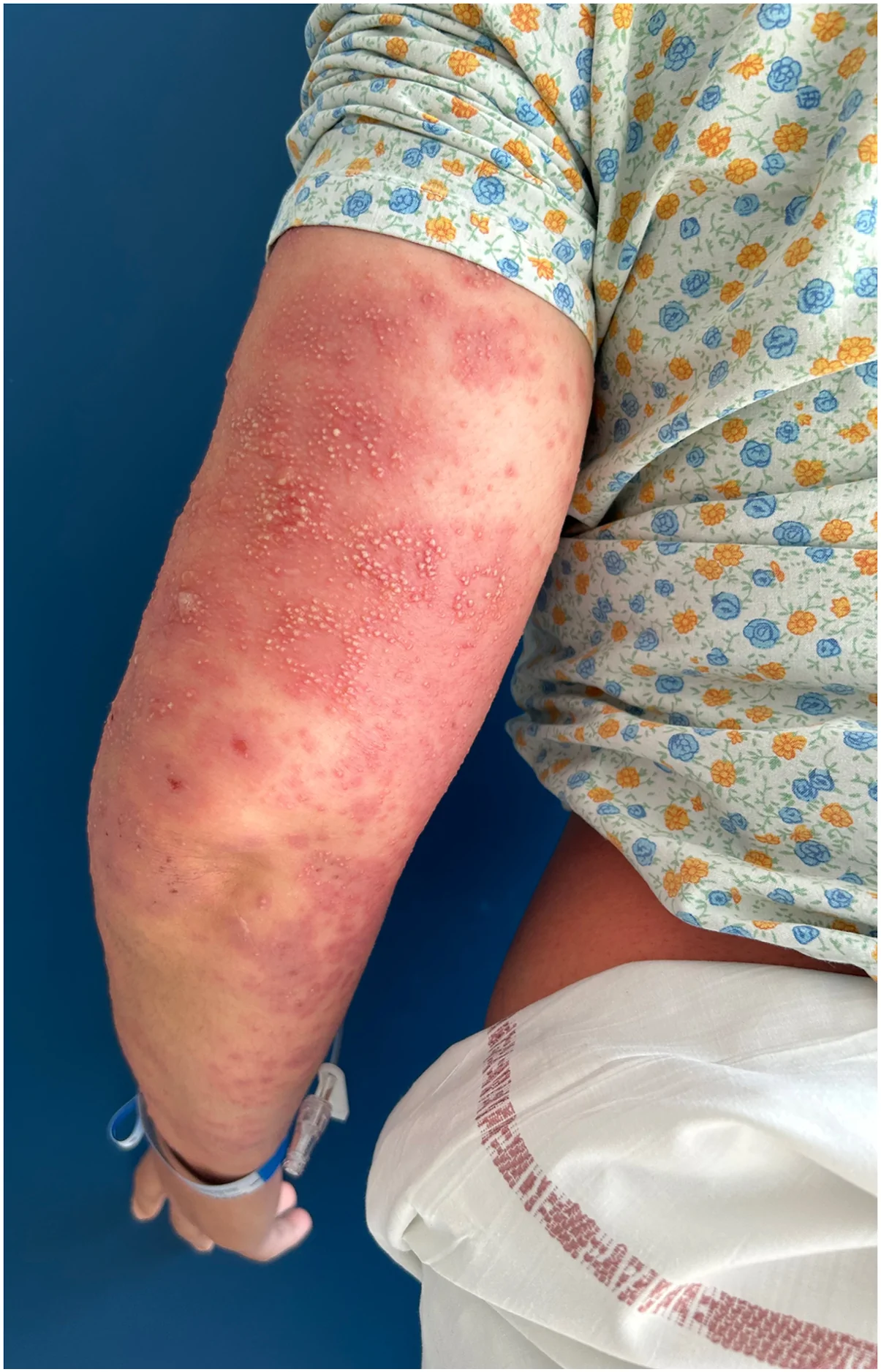
Clinical aspect of the upper limbs showing numerous small, disseminated pustules overlying a highly inflammatory erythematous background.

**Fig 3:**
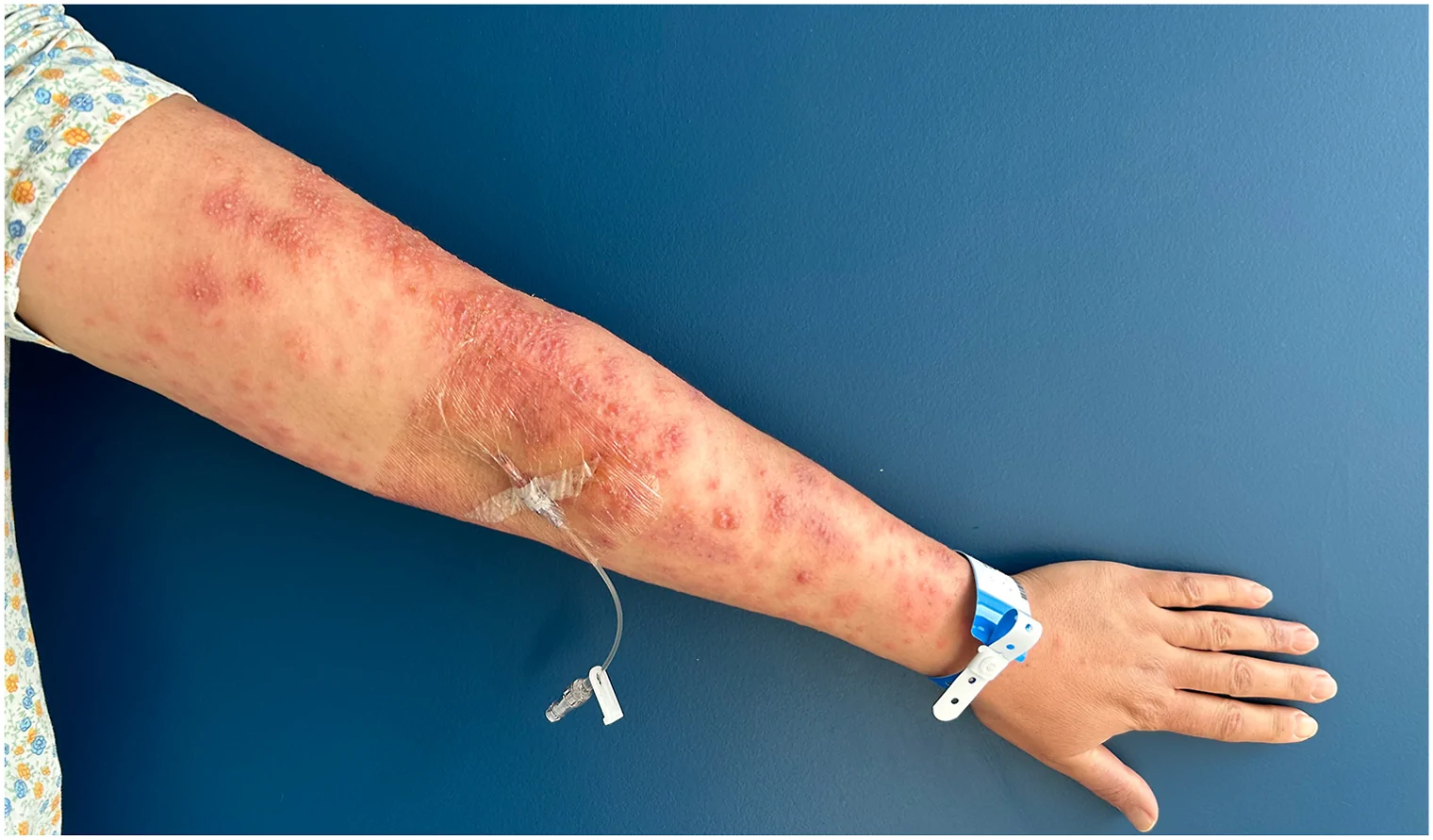
Polymorphic eruption on the left arm characterized by a combination of pustules, vesicles, and larger bullae on a highly inflammatory background.

Within 24 hours, the condition worsened, with fever (up to 38.9 °C), malaise, and vomiting. Laboratory investigations revealed a significant inflammatory response with a C-reactive protein level of 210 mg/L, without leukocytosis. However, a profound lymphopenia of 0.6 G/L was observed, unexplained by any identifiable hematological cause. Renal and liver function tests were normal.

Extensive investigations were negative, including serologies for human immunodeficiency virus (HIV), hepatitis B virus, hepatitis C virus, varicella-zoster virus, cytomegalovirus, Epstein-Barr virus, and parvovirus B19, as well as autoimmune screening (antinuclear antibodies and antineutrophil cytoplasmic antibodies) Serum protein electrophoresis and immunoglobulin levels were normal, with no evidence of malignancy or inflammatory bowel disease.

*M pneumoniae* IgM was positive (14; threshold >12), in the context of a mild cough with a normal chest radiograph, leading to treatment with azithromycin (500 mg on day 1, then 250 mg/day for 6 days). *Mycoplasma* polymerase chain reaction (PCR) was not performed, and the absence of IgG seroconversion at 1 month precluded definitive confirmation.

Skin biopsy showed intraepidermal and subepidermal bullous cleavage. The blister roof was frequently altered and contained neutrophilic micropustules. Intraepidermal pustules, some in the deeper epidermis, were observed with an abundance of neutrophils and eosinophils (Fig 4, *A*). The dermis showed a dense superficial and mid-dermal inflammatory infiltrate, both perivascular and interstitial, composed of neutrophils and numerous eosinophils (Fig 4, *B*). Direct immunofluorescence revealed intraepidermal intercellular IgA and C3 deposits (Fig 4, *C*), without IgG, IgM, or linear basement-membrane-zone staining. Indirect immunofluorescence on monkey esophagus confirmed circulating IgA antibodies targeting the intercellular substance, whereas circulating IgG was negative. Antidesmoglein 1 and 3 enzyme-linked immunosorbent assays were negative, and assays for antidesmocollins were unavailable at our facility.

**Fig 4:**
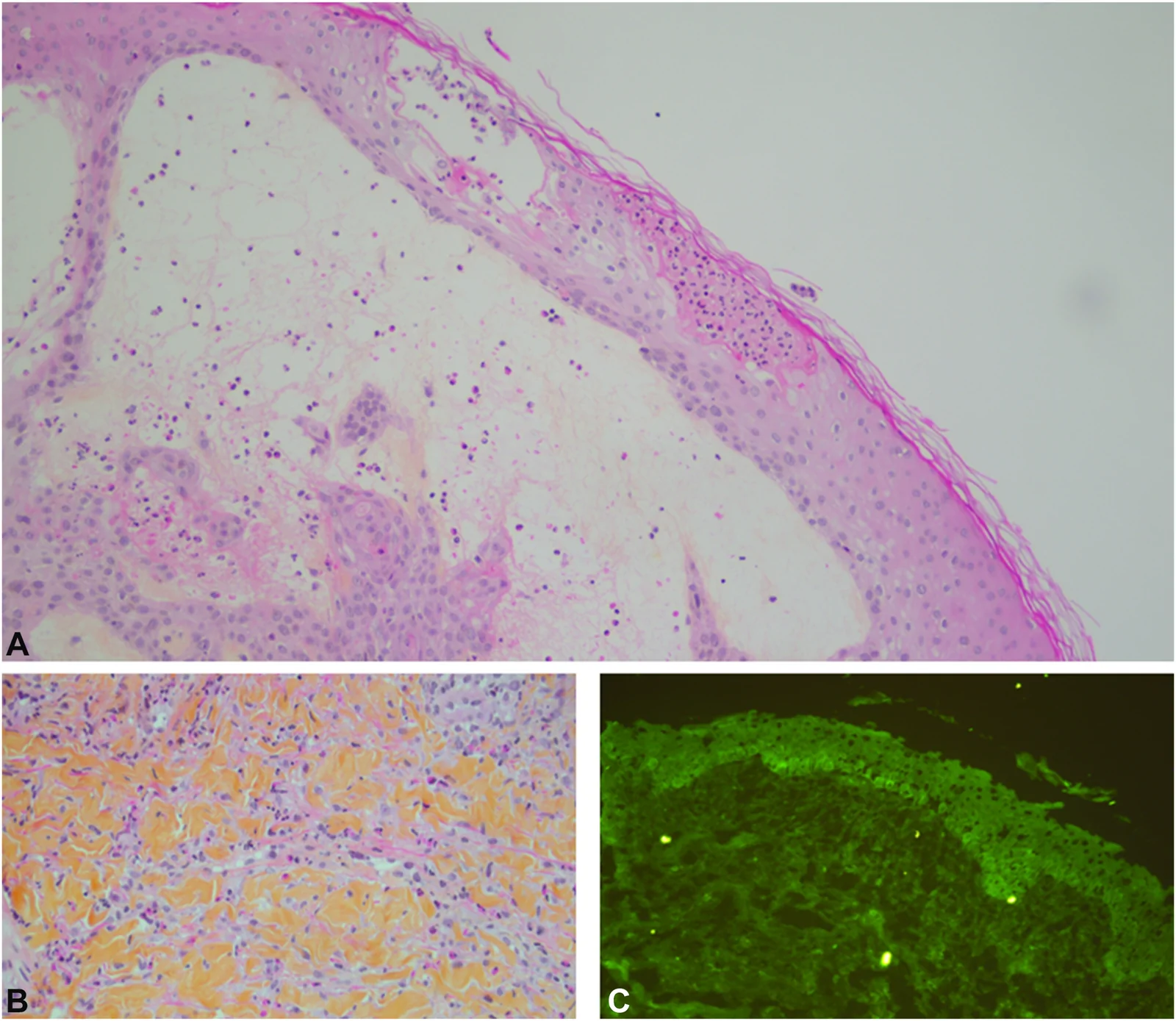
**A,** Hematoxylin and eosin stain (original magnification ×100) showing intraepidermal and subepidermal cleavage with neutrophilic micropustules in the blister roof. **B,** Higher magnification (×400) revealing a dense dermal infiltrate with numerous eosinophils and neutrophils. **C,** Direct immunofluorescence demonstrating intercellular IgA deposits throughout the epidermis.

A diagnosis of IEN-type IgA pemphigus was made.

Given the inflammatory nature of the lesions, treatment with high-potency topical corticosteroids (clobetasol propionate 0.05% cream) was initiated while awaiting glucose-6-phosphate dehydrogenase level results, prior to the planned introduction of dapsone. However, due to the dramatic clinical improvement within 2 weeks, dapsone was ultimately not introduced, and the patient continued with topical therapy alone. After a total treatment duration of 1 month, all topical therapy was discontinued.

The patient remains in complete remission without any recurrence at 6 months follow-up.

## Discussion

IgA pemphigus is frequently associated with systemic comorbidities such as monoclonal gammopathies, internal malignancies, and inflammatory bowel disease. IgA pemphigus temporally associated with an acute bacterial infection has, to our knowledge, rarely been reported, although cases linked to HIV infection and COVID-19 vaccination suggest that externally triggered forms may follow a more benign course.^1^, ^2^, ^3^

Several inflammatory pustular and bullous disorders were considered. Acute generalized exanthematous pustulosis (AGEP) was unlikely given the absence of a recent culprit drug, the tense vesicles and bullae accompanying the pustules, the absence of leukocytosis, and the intercellular IgA deposits, which are not features of AGEP. Linear IgA bullous dermatosis was excluded by the intercellular rather than linear basement-membrane-zone IgA pattern. Bullous Sweet syndrome lacks acantholytic intraepidermal pustulation and intercellular IgA. A primary infectious bullous eruption was excluded by negative lesional bacterial cultures. Finally, despite positive *M pneumoniae* IgM, *Mycoplasma*-induced rash and mucositis and erythema multiforme-like eruption were considered unlikely given the absence of mucosal involvement, the bullo-pustular rather than targetoid morphology, and the intercellular IgA deposits.

The acute systemic presentation, combined with initial *M pneumoniae* IgM seropositivity, raises the possibility of an infectious trigger, as *M pneumoniae* can induce dysregulated immune responses through polyclonal B-cell activation and molecular mimicry and has been linked to IgA-mediated phenomena such as IgA vasculitis.^4^, ^5^

This inference nonetheless rests on limited evidence: respiratory symptoms were minimal, chest imaging was limited to a plain radiograph, and *M pneumoniae* PCR was not performed. Serological support relied on a positive IgM, which can be falsely positive; paired sera obtained 1 month apart showed persistent IgM without IgG seroconversion. While a blunted humoral response related to the profound lymphopenia could contribute to this absent seroconversion,^6^ the overall profile does not confirm acute infection. Whether *M pneumoniae* acted as a genuine trigger or was a concurrent finding therefore remains unproven.

The therapeutic course is remarkable. Current guidelines recommend dapsone as first-line therapy, often long-term and with frequent relapses on dose reduction; sustained remission with topical corticosteroid monotherapy alone has rarely been reported.^7^ The dramatic response within 2 weeks of clobetasol propionate, with sustained remission after discontinuation at 1 month, suggests that once the triggering stimulus is cleared the pathogenic IgA response may resolve without long-term immunomodulation.

This raises a practical question: should dapsone be initiated systematically in all presentations of IgA pemphigus, or should an expectant strategy be considered first in selected patients with acute, systemically inflammatory presentations and a potential identifiable trigger? A single case cannot answer this, but our observation argues for an individualized approach.

## Conclusion

This case describes an unusual, acute, systemically inflammatory presentation of IEN-type IgA pemphigus with a striking response to topical corticosteroid monotherapy. This single observation raises the possibility of a transient, trigger-associated presentation of IgA pemphigus, in which a self-limited autoimmune response resolves completely without systemic treatment. This hypothesis requires confirmation in further cases. Should this pattern be confirmed, an initial conservative approach might be considered in selected patients with acute presentations and systemic symptoms, potentially sparing them systemic toxicities.

## Conflicts of interest

None disclosed.
